# Efficacy and safety of Kanglaite injection combined with chemotherapy for women breast cancer: A protocol for systematic review and meta-analysis of randomized clinical trials

**DOI:** 10.1097/MD.0000000000026245

**Published:** 2021-06-04

**Authors:** Shengli Cheng, Biao Qu, Xiaoxia Qiu, Nannan Li, Xiaoli Wang, Jiahu Hao

**Affiliations:** a Anhui Medical University, School of Public Health; b The First Affiliated Hospital of University of Science and Technology of China, Anhui Provincial Hospital, Hefei, Anhui Province; c Macau University of Science and Technology, Taipa, Macau (SAR); d Shulan (Hangzhou) Hospital Affiliated to Zhejiang Shuren University Shulan International Medical College, Hangzhou, Zhejiang Province, China; e Qinghai Province Cardiovascular and Cerebrovascdular Disease Specialist Hospital, Xining, Qinghai Province.

**Keywords:** chemotherapy, Kanglaite injection, meta-analysis, systematic review, women breast cancer

## Abstract

**Background:**

Breast cancer was the second cause of cancer death and approximately accounted for 30% of all newly diagnosed cancer in American women. Adjuvant chemotherapy is the preferred treatment approach for breast patients. Kanglaite injection (KI) was commonly used as adjuvant chemotherapy combined with chemotherapy for women breast cancer which could increase chemotherapy efficacy and alleviate chemotherapy drugs induced adverse events, however, the efficacy and safety for KI combined western medicine remains controversial. Thus, we conducted this protocol of systematic review and meta-analysis to estimate the efficacy and safety of KI combined with western medicine for women breast cancer.

**Methods:**

This study will search electronic database included English medicals databases and Chinese databased up to May 2021. The main outcomes of this study include clinical efficacy rate. Adverse reaction rate, Karnofsky Performance Status and immune function were defined as the secondary outcomes.

**Results:**

This protocol study will comprehensively evaluate the efficacy and safety of KI combined with chemotherapy for women breast cancer.

**Conclusion:**

This protocol for systematic review and meta-analysis will evaluate the efficacy and safety of KI combined with chemotherapy for women breast cancer, aiming to provide optimal therapy for women breast cancer patients.

## 1 Introduction

Breast cancer was the second cause of carcinoma death and the most common malignancy in women worldwide which developed from mammary gland epithelial tissue.^[1–3]^ It is estimated that about 2.1 million new cases were diagnosed with breast cancer and 626,679 women died for breast cancer in 2018.^[4,5]^ The global breast cancer incidence has been rising with annual increases of 3.1% and approximately accounts for 30% of all newly diagnosed cancer in American women.^[6,7]^ In addition, the mortality of breast cancer in South America, Africa, and Asia is also still increasing, partly because of a lack of access to advanced diagnosis and therapy^[8]^ which seriously threatens women's physical and mental health. It is curable for patients with early-stage, nonmetastatic breast cancer (70%–80%), while advanced breast cancer with organ metastases is considered incurable with currently available therapies.^[5]^

Management of breast cancer involved multidisciplinary approaches, such as locoregional (surgery and radiation therapy) and systemic therapy (chemotherapy, immunotherapy, etc). Adjuvant chemotherapy is the preferred treatment approach for breast cancer patients.^[9–11]^ Besides, adjuvant chemotherapy potentially enhanced the probability of tumor resectability and breast conservation, patients could survive longer or even overall after a partly or completely responded adjuvant therapy.^[12–14]^ However, the decreased immune function, increased toxicity, and adverse reactions after chemotherapy are not satisfying. Therefore, it is essential to find a better therapy to increase chemotherapy efficacy and reduce adverse reactions. Traditional Chinese medicines have been widely combined with chemotherapy drugs to increase chemotherapy efficacy and alleviate chemoradiotherapy drugs induced adverse events.^[15–18]^ Kanglaite injection (KI) is one of the antitumor Chinese patent medicines approved by the State Food and Drug Administration of China (Drug Approval Number: Z10970091) for adjuvant chemotherapy.^[19]^ KI was extracted from a traditional Chinese medicinal plant, Semen cocis, which could kill cancer cells, improve patient immune function and reduce adverse effects.^[20–22]^ Increased clinical trials have explored the effectiveness of KI as an adjuvant drug to western medicine chemotherapy for breast cancer patients, however, the clinical outcomes were discrepant and the conclusion remained controversial. Some randomized clinical trials (RCT) showed that combined therapy improved clinical efficacy and reduced toxicity and adverse effects, while some clinical trials exhibited no significant effects.^[23,24]^ The efficacy and safety of KI combined with western medicine for breast cancer patients have never been systematically evaluated. Thus, it is essential to estimate the efficacy and safety of KI combined with western medicine for breast cancer, aiming to provide optimal therapy for women breast cancer patients.

## 2 Methods

### 2.1 Study registration

The protocol of the systematic review has been registered (OSF Preregisration number: DOI 10.17605/OSF.IO/CN9J8). This systematic review and meta-analysis protocol will follow PRISMA guidelines statement guidelines.^[25]^

### 2.2 Criteria for eligible studies selection

#### 2.2.1 Types of studies

The randomized controlled trials study of KI combined with western medicine vs western medicine are eligible regardless of publication language, year, and status.

#### 2.2.2 Types of participants

RCT included patients were diagnosed with esophageal carcinoma (18 years or older), any of the degree and possible complications. One of the following indexes should be included: clinical efficacy rate, Karnofsky Performance Status, immune function, and adverse reaction rate.

#### 2.2.3 Type of interventions

Patients in the control group were treated with chemotherapy drugs. The treatment group was treated with conventional chemotherapy drugs plus KI.

#### 2.2.4 Outcome measures

The main outcomes of this study include clinical efficacy rate. Adverse reaction rate, Karnofsky Performance Status, and immune function were defined as the secondary outcomes.

### 2.3 Data collection and analysis

#### 2.3.1 Electronic searches

To identify all relevant clinical studies, we will search the following electronic databases from their inception to May 2021. Electronic database included 4 English medicals databases and 5 Chinese databases: PubMed, Embase, MEDLINE, Cochrane Library, China National Knowledge Infrastructure (CNKI) database, Wanfang Database, Chinese Scientific Journals Database, Chinese Biomedical Literature database, and Chinese Science Citation Database. Our search strategy for PubMed is displayed in Table 1.

**Table 1:** Search strategy in PubMed database.

Number	Search terms
#1	Breast Neoplasm[MeSH]
#2	Neoplasm, Breast[Title/Abstract]
#3	Breast Tumor[Title/Abstract]
#4	Breast Tumors[Title/Abstract]
#5	Tumor, Breast[Title/Abstract]
#6	Tumors, Breast[Title/Abstract]
#7	Neoplasms, Breast[Title/Abstract]
#8	Breast Cancer[Title/Abstract]
#9	Cancer, Breast[Title/Abstract]
#10	Mammary Cancer[Title/Abstract]
#11	Cancer, Mammary[Title/Abstract]
#12	Cancers, Mammary[Title/Abstract]
#13	Mammary Cancers[Title/Abstract]
#14	Malignant Neoplasm of Breast[Title/Abstract]
#16	Breast Malignant Neoplasm[Title/Abstract]
#17	Breast Malignant Neoplasms[Title/Abstract]
#18	Malignant Tumor of Breast[Title/Abstract]
#19	Breast Malignant Tumor[Title/Abstract]
#20	Breast Malignant Tumors[Title/Abstract]
#21	Cancer of Breast[Title/Abstract]
#22	Cancer of the Breast[Title/Abstract]
#23	Mammary Carcinoma[Title/Abstract]
#24	Human Carcinoma[Title/Abstract]
#25	Human Mammary Carcinomas[Title/Abstract]
#26	Mammary Carcinomas, Human[Title/Abstract]
#27	or/1–26
#28	Kanglaite[Title/Abstract]
#29	Therapy, Drug[Title/Abstract]
#30	Drug Therapies[Title/Abstract]
#31	Chemotherapy[Title/Abstract]
#32	Chemotherapies[Title/Abstract]
#33	or/29–33
#34	#27 and #28 and #33

#### 2.3.2 Studies selection

**Figure 1:**
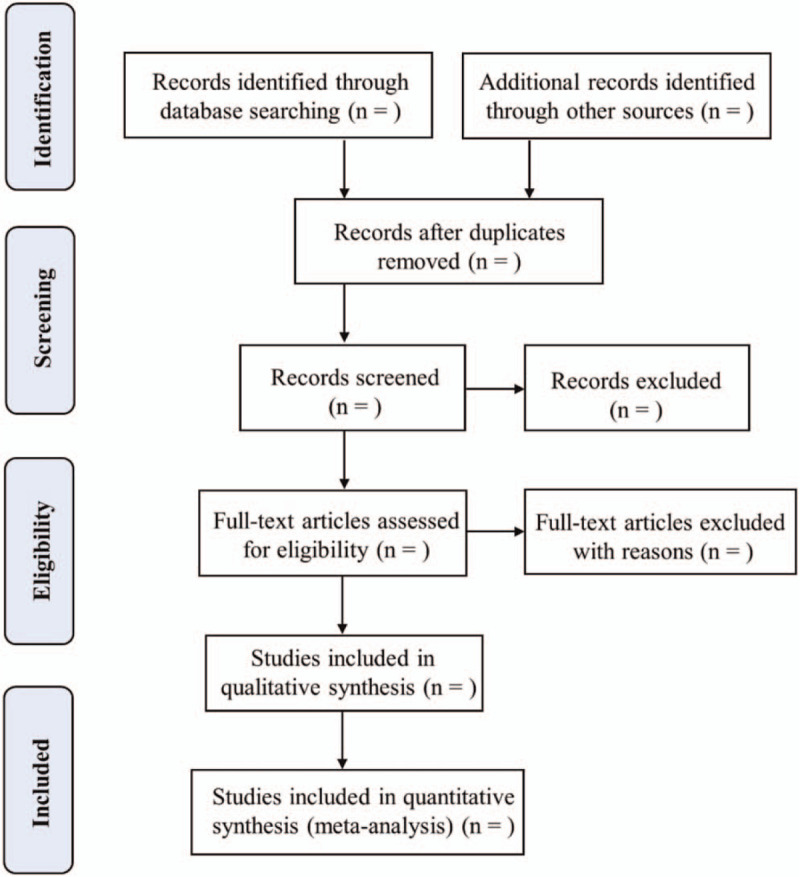
Flow chart of this study selection process.

Two independent researchers (SLC and NNL) will independently perform the selection of titles and abstracts of all the relevant articles. Full text will be download for further selection based on inclusion and exclusion criteria. All discussions will be conducted for disagreement. Flow chart of this study selection (Fig. 1) will exhibit the data screening process of this study.

#### 2.3.3 Data collection and management

Two reviewers (SLC and XLW) will independently screen the contained RCTs and extract data according to Cochrane handbook of Systematic Reviews of Interventions. If a trial result was ambiguous or partial data would be resolved by contacting corresponding article authors. If there is a conflict, a third reviewer will participate in the discussion (QB).

#### 2.3.4 Assessment of risk of bias

Reviews evaluated the relevant clinical trials study according to the guidelines of Cochrane handbook of Systematic Reviews of Interventions.^[26]^ Criteria will be adopted to value bias risks via selection bias, performance bias, detection bias, attrition bias, reporting bias and other bias. The estimate will be divided into 3 levels: “low risk,” “high risk,” or “unclear risk.” Conflict will be resolved by the third reviewer (JHH).

#### 2.3.5 Data analysis

Review Manager 5.3 (Copenhagen: The Nordic Cochrane Centre, The Cochrane Collaboration, 2014), the Comprehensive Meta-Analysis (CMA) 3.0 will be used to combine trials study. Continuous data displayed as weighted mean difference (WMD) and dichotomous outcome as risk ratio (RR), risk difference (RD) or odds ratio (OR) with their 95% confidence intervals (CI). Chi^2^ test and *I*^2^ statistic will be performed to value statistical heterogeneity. When *I*^2^ > 50%, or *P* < .1, substantial heterogeneity will be considered to play a role, and the random-effects model will be applied to estimate the summary RR (OR or RD), WMD and 95% CI; otherwise, a fixed-effects model will be selected. What is more, sensitivity analysis will be used to verify the robustness of the results.

#### 2.3.6 Patient and public involvement

This meta-analysis was performed by previously published data, thus patient and public content will not be included in this study.

#### 2.3.7 Ethics and dissemination

Ethical approval was not essential as this article was a protocol for systematic review and meta-analysis. We prepared to display this protocol in a journal or conference presentation.

#### 2.3.8 Evidence assessed

The quality of evidence for this study will be evaluated by “Grades of Recommendations Assessment, Development and Evaluation (GRADE)” standard developed by the World Health Organization and international organizations.

## 3 Discussion

To the best of our knowledge, this is the first study to date to comprehensively compare the efficacy and feasibility of KI combined with western medicine for women breast cancer. We conducted this systematic review and meta-analysis according to PRISMA guidelines to provide optimal therapy for women breast cancer patients.

## Author contributions

**Conceptualization:** Shengli Cheng, Nannan Li, Xiaoxia Qiu, Biao Qu.

**Data curation:** Shengli Cheng, Xiaoli Wang, Biao Qu, Jiahu Hao.

**Formal analysis:** Shengli Cheng, Nannan Li, Biao Qu, Jiahu Hao.

**Methodology:** Shengli Cheng, Xiaoli Wang, Biao Qu.

**Software:** Shengli Cheng, Nannan Li.

**Visualization:** Biao Qu, Jiahu Hao.

**Writing original draft:** Shengli Cheng, Nannan Li.
